# Short-term load forecasting using a metaheuristic optimized temporal fusion transformer with decomposition technique

**DOI:** 10.3389/frai.2026.1797906

**Published:** 2026-05-11

**Authors:** Radhika Chandrasekaran, Senthil Kumar Paramasivan

**Affiliations:** School of Computer Science Engineering and Information Systems, Vellore Institute of Technology, Vellore, Tamil Nadu, India

**Keywords:** attention mechanism, decomposition, deep learning, gated residual network, optimization, temporal fusion transformer

## Abstract

Short-term load forecasting plays a vital role in today's modern life to ensure the balance between energy demand and supply. Dynamic variations in weather and electricity consumption patterns can significantly influence load patterns, resulting in complex modeling and challenging forecasting accuracy due to non-stationary, non-linear patterns. Traditional statistical methods are simple and interpretable; however, they struggle to capture temporal and non-linear dependencies. Although machine learning can address these challenges, it struggles with long-range temporal dependency. In contrast, deep learning models can automatically extract temporal patterns and relevant features from raw data. This study proposes a Temporal Fusion Transformer (TFT) based on Multivariate Variational Mode Decomposition (MVMD) and optimized with the GOAT Optimization Algorithm (GOA). The deep architecture of the transformer model is considered an alternative owing to its self-attention mechanism, its efficiency in capturing long sequences, its parallel processing capabilities, and its interpretability through attention weights, making it suitable for multivariate short-term load forecasting. The significant benefit of combining MVMD with TFT is that it enhances feature extraction by decomposing complex multivariate time series data into components with different frequencies and reduces noise. The MVMD addresses the non-stationary and non-linear challenges of energy load data with reduced complexity and interpretability. Furthermore, the GOAT Optimization Algorithm was incorporated for hyperparameter tuning of the TFT model to enhance its performance. The error in the model is evaluated using mean absolute error (MAE), mean absolute percentage error (MAPE), root mean square error (RMSE), and symmetric mean absolute percentage error (sMAPE). The proposed model outperforms other comparison models. Finally, the model is interpreted using SHapley Additive exPlanation (SHAP) analysis to understand the impact of features on the model's prediction.

## 1 Introduction

Over the next 20 years, due to the global population growth, energy consumption is likely to increase by 48%. At present, approximately 80% of the global demand is seen to be for fossil fuels, which significantly challenges the sustainability (Moodley, 2021; Goren et al., 2023). Short-term load forecasting is essential for improving power system operational efficiency by accurately predicting energy demand, and the time horizon for predicting electricity demand ranges from a few minutes to several days (Eren and Küçükdemiral, 2024). Precise forecasting is essential for minimizing operational costs, optimizing generation schedules, maintaining grid stability, and efficiently allocating energy resources in a dynamic, complex landscape (Han et al., 2025). Short-term load forecasting poses significant challenges due to the complexity and volatility of data, temporal dependencies, and patterns that impact prediction accuracy. The influence of external variables such as weather, consumption behavior, socioeconomic factors, calendar effects, and random disturbances will be reflected in an accurate load prediction. In addition, the average load consumption and maximum load demand are determined by electricity costs and the degree of industrialization. The fluctuation in energy demand will influence data-driven decisions related to unit commitment, maintenance, and economic dispatch.

Short-term load forecasting employs various forecasting techniques, including statistical, machine learning, deep learning, and hybrid models, to capture temporal dependencies and other relevant aspects (Liu et al., 2023). The conventional statistical models, such as Autoregressive Moving Average (ARMA) (Huang and Shih, 2003), Autoregressive Integrated Moving Average (ARIMA) model (Alberg and Last, 2018), Seasonal Autoregressive Integrated Moving Average (SARIMA) model (Bozkurt et al., 2017), exponential smoothing (Taylor and McSharry, 2007), and the Kalman filtered Monte Carlo method (Owusu et al., 2023), are effective with stable linear patterns. However, they struggle to capture non-linear dependencies, are sensitive to outliers, and are prone to overfitting, limiting their ability to generalize unseen data effectively.

As fluctuations and data complexity increase, they exacerbate the inadequacy of statistical approaches in handling non-linear and temporal dependencies. Machine learning models can address these challenges by capturing the intricate patterns and non-linear relationships to improve model performance. The machine learning models, such as K-Means and Support Vector Machines (SVMs) (Dong et al., 2022), Fuzzy c-mean clustering with random forest and Deep Neural Networks (DNNs) (Liu et al., 2021), K-means and K-nearest neighbors (KNN) (Hnin et al., 2024), and self-organizing map (Lee et al., 2019) can capture intricate patterns and non-linear relationships to improve accuracy. Models such as random forest and gradient boosting automatically reduce noise by increasing forecasting accuracy (Wang et al., 2018). A Bayesian neural network provides probabilistic estimates, including point forecasts, and valuable insights for decision-making (Hippert and Taylor, 2010). Insufficient data can lead to inaccurate predictions, and traditional models may struggle with high-dimensional, unstructured data.

Deep learning has addressed the limitations of machine learning effectively. From the raw data, it learns automatically to capture the non-linear relationships and extract relevant features from large datasets, thereby improving peak load prediction. The deep learning models, such as Recurrent Neural Networks (RNNs) (Zhang et al., 2018), Long Short-Term Memory (LSTM) (Majeed et al., 2025; Ijaz et al., 2022), Bidirectional Long Short-Term Memory (BiLSTM) (Hu et al., 2023), Convolutional Neural Network (CNN)-LSTM (Li and Shi, 2025), Bidirectional Gated Recurrent Unit (BiGRU) and stacked autoencoder (Dong et al., 2025) can capture the long-term temporal dependencies in high-dimensional data. The conventional deep learning models also face issues related to highly sensitive data quality, capturing multiscale temporal patterns, computational cost, generalization, and a lack of interpretability. The transformer model can handle the sequential processing bottleneck by parallelization, enabling parallel processing with a self-attention mechanism. The transformer models, such as temporal-augmented transformers, learn the temporal relationships with the previous data to capture the dynamics of non-linear sequences to support short-term load forecasting (Ran et al., 2023; Zhang G. et al., 2022).

The challenges in standalone models can be addressed by combining them into a hybrid model for forecasting. To reduce the complexity of time series and increase robustness, a hybrid model is needed. Some hybrid models, such as Seq2Seq LSTM with attention (Buratto et al., 2024), Multiple Linear Regression/Multilayer (MLR) and LSTM (Li et al., 2020), and Bidirectional RNN with Deep Belief Network DBN (Tang et al., 2019), can address the deficiencies of standalone models and achieve better outcomes. The hybrid models can also be a combination of deep learning models with decomposition techniques and feature extraction to balance the prediction accuracy in short-term load forecasting.

The integration of decomposition techniques, such as Empirical Mode Decomposition (EMD), Ensemble Empirical Mode Decomposition (EEMD), Complete Ensemble Empirical Mode Decomposition (CEEMDAN), and Variational Mode Decomposition (VMD), with deep learning reduces model complexity. Some hybrid models, such as VMD and wavelet-based CNN (Ahajjam et al., 2022), two-layer decomposition with dynamic optimal ensemble learning (Lin et al., 2025), VMD with stacking model fusion (Zhang Q. et al., 2022), EMD with BiLSTM (Mounir et al., 2023), EEMD with Sparrow Search Algorithm (SSA) and BiLSTM (Zhang, 2025), and VMD LSTM with a Bayesian Optimization Algorithm (He et al., 2019), improve the accuracy of the model. Transformer models, including transformer-based fusion on CNN-BiGRU (Xie et al., 2025), Sparse transformer (Chan and Yeo, 2024), transformer for smart grids (Wang and Zhao, 2021), and a federated model agnostic meta learning for distribution transformer (Feng et al., 2024), support improving the model performance. The combination of ICEEMDAN, fuzzy entropy with CNN, BILSTM, and an improved Sparrow Search Algorithm can handle incomplete feature extraction and long-term dependencies (Tang et al., 2025). An improved gray wolf genetic algorithm with level processing improves the prediction accuracy (Li, 2025). A Temporal Convolutional Network (TCN) with self-attention and BiLSTM integration is used to capture long-term dependencies and enable robust temporal relationships (Hu et al., 2025). A dynamic Particle Swarm Optimization (PSO) with a least-squares Support Vector Machine to dynamically tune parameters and achieve robust adaptability (Ji et al., 2025).

Multi-region hourly electricity load data across different horizons are forecasted using a multitask graph convolutional network combined with attention-based seasonal decomposition (Zhang et al., 2024). A combination of CEEMDAN, TCN, AutoLSTM, and a cross-stitch network for next-day and next-week predictions. The cross-stitch network for information exchange outperforms standalone models such as TCN and LSTM. The limitation of this model is its reliance on single-region data, with no external features, such as weather, prices, and socioeconomic factors (Sakib and Mustajab, 2025). An exponential smoothing with an Exponential Smoothing and Dilated Recurrent Neural Network (ES-dRNN) for one-day-ahead forecasting and hourly granularity in short-term load forecasting. This model focuses on univariate data and is tested only on a European dataset. No external features were considered (Smyl et al., 2023). To improve the generalization and performance of 24-h prediction, a transformer-based model is used for short-term load forecasting; however, it limits performance across regions (Ahmad et al., 2025). An LSTM Transformer Encoder model for single-step forecasting with application-level load data for each household. This model has no external features, and only US residential data were tested; therefore, generalization issues may arise (Pentsos et al., 2025). A spatial- and temporal-based transformer for short-term load forecasting captures spatial correlations without prior geographic information. However, this model may face scalability issues in some regions (Zhao et al., 2023). A combination of a multi-head CNN and a BiLSTM with lag parameters can handle long-term dependencies (Han and Zeng, 2024). Based on prior studies, it is observed that the majority of the models focus only on univariate data, mostly historical load data, and a limited region. Considering external variables such as meteorological data, price, and economic data will be more challenging. Training the model on univariate data from a limited region leads to generalization issues.

### 1.1 Related study

Despite recent advancements in short-term load forecasting, they still face challenges such as sensitivity to non-stationary data, limitations in robust multivariate settings, interpretability, generalization, and hyperparameter tuning. The detailed comparison of hybrid models to highlight the limitations of existing methods is depicted in Table 1.

**Table 1:** Comparison of hybrid approaches in short-term load forecasting.

References	Year	Model used	Decomposition method	Optimization technique	Key limitations
Fan et al. (2025)	2025	Bidirectional temporal convolutional network (BiTCN) Long short-term memory (LSTM)	–	Improved Salp swarm algorithm (ISSA)	Lack of decomposition and insufficient interpretability
Sakib and Mustajab (2025)	2025	Temporal convolutional network (TCN) AutoLSTM Cross-stitch network	Complete ensemble empirical mode decomposition (CEEMDAN)	Automatic hyperparameter tuning LSTM (AutoLSTM)	Lack of external hyperparameter tuning. Informative intrinsic mode functions (IMFs) selection is crucial
Li et al. (2024)	2024	Temporal convolutional network (TCN)–gated recurrent unit (GRU)	Improved CEEMDAN (ICEEMDAN) Adaptive variational mode decomposition (AVMD)	–	Lack of optimization, difficulty in capturing global dependencies, and limited interpretability
Tie et al. (2025)	2025	Convolutional neural network (CNN) Bidirectional BiGRU (BiGRU) Autoformer (CA-BiGRU + autoformer)	Built in autoformer (trend–seasonal)	–	Lack of optimization and interpretability
Yang et al. (2025)	2025	Multi-granularity—autoformer Multi-granularity autocorrelation attention mechanism (MG-ACAM) Query-key mechanism	Series decomposition	Gradient-based optimization	Lack of external influential factors Limited generalization
Qu et al. (2025)	2025	Patch time series transformer (PatchTST)—progressive layered extraction (PLE)	Triangulation topology aggregation optimizer (TTAO)–variational mode decomposition (VMD)	TTAO	Optimization applied to decomposition only. limited global tuning
Yue et al. (2022)	2022	LSTM	Ensemble empirical mode decomposition (EEMD)	Bayesian optimization algorithm (BOA)	Limitation in capturing global dependencies
He et al. (2024)	2024	TimesNet Crossformer LSTM	–	–	Lack of optimization + decomposition, and interpretability
Brahim et al. (2025)	2025	Modified transformer–bidirectional LSTM–domain adaptation forecaster (TF-BiLSTM DAF)	–	Manual trial and error. Adam	Lack of robustness and reliability
Tan et al. (2024)	2024	Improved temporal convolutional network (ITCN) Improved time series transformer (ITST)	Adaptive median filter—empirical mode decomposition (AMF-EMD)	–	No hyperparameter tuning was performed using optimization techniques Limited interpretability

Although existing methods used in short term load forecasting has significant advancement it struggles with certain limitations as discussed below,

- Traditional deep learning models struggle to capture the non-stationarity and non-linearity characteristics of load data efficiently. It lacks interpretability for complex deep learning predictions and fails to incorporate attention mechanisms to selectively highlight relevant past information.
- The decomposition methods used in hybrid models are mostly univariate analysis and are sensitive to complex multivariate and non-stationary features.
- The traditional hyperparameter tuning methods, including manual, grid, and random, are insufficient for complex architectures and high-dimensional spaces, as they suffer from computational overhead and noise.

To address these limitations, this study proposes a unified Multivariate Variational Mode Decomposition-Temporal Fusion Transformer-GOAT Optimization Algorithm (MVMD-TFT-GOA) framework with enriching feature representation through multivariate decomposition to handle irregular and complex patterns addressing non-stationary and non-linearity, enabling granular interpretation of deep learning forecasts at multiple scales with TFT to capture temporal dependencies through its internal gating structure and multi-head self-attention mechanism, and demonstrating a robust methodology for optimizing complex neural networks with GOA to find global solution for TFT through efficient exploration and exploitation balance by avoiding premature convergence thereby pushing the boundaries of accuracy and enhancing the explainability in short-term load forecasting with SHapley Additive exPlanation (SHAP).

### 1.2 Significant contribution of this study

The main contribution of this proposed study is summarized as follows:

- A novel unified framework is developed, integrating MVMD decomposition with TFT to capture complex temporal patterns effectively.
- Enhancing forecasting accuracy with low prediction errors by effectively handling non-stationary load patterns with MVMD.
- Incorporation of the GOAT Optimization Algorithm (GOA) improves model stability and convergence.
- Achieved reliable performance with several evaluation metrics and enhanced the interpretability with SHAP analysis.

### 1.3 Pipeline of proposed MVMD-TFT-GOA framework

**Figure 1:**
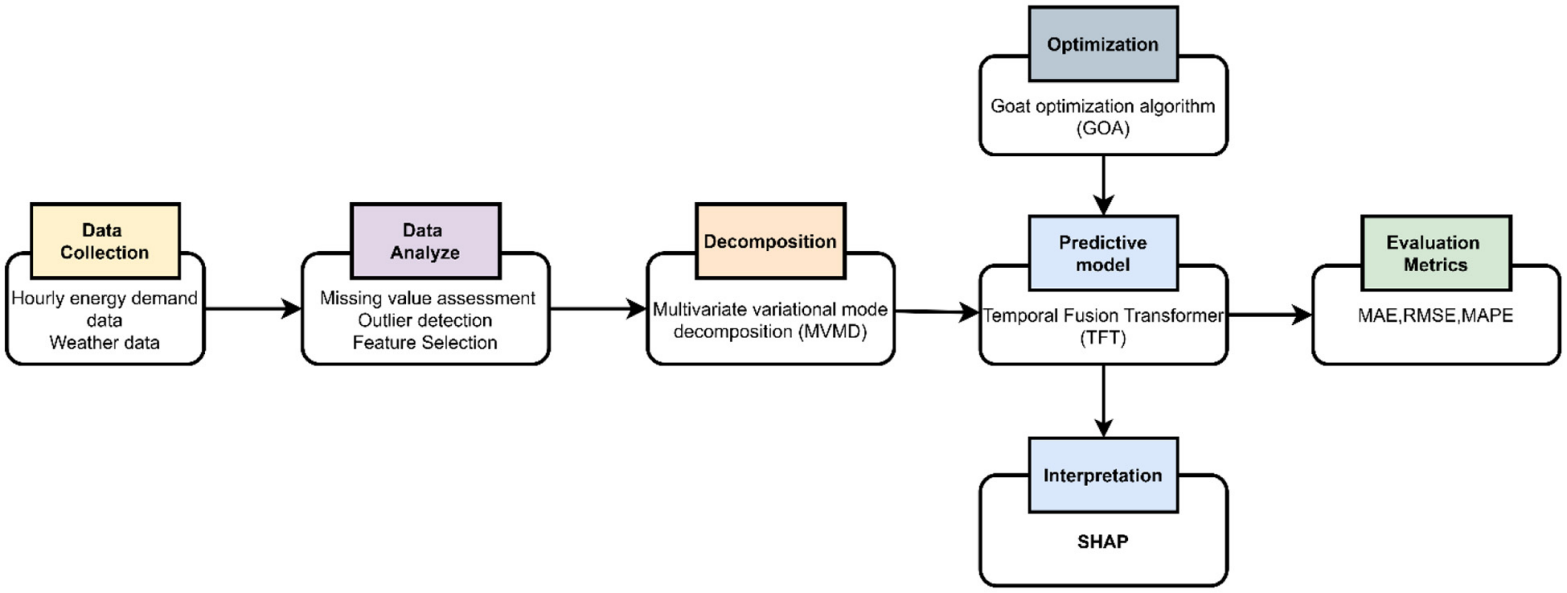
Schematic pipeline of MVMD-TFT-GOA.

The proposed model (MVMD-TFT-GOA) schematic workflow is depicted in Figure 1.

- The data preprocessing framework combines missing value assessment, interquartile range (IQR)-based statistical outlier detection, min-max normalization for feature scaling, and filter-based feature selection with PCC for significant feature ranking to ensure high-quality feature information for subsequent modeling.
- The key features were decomposed using Multivariate Variational Mode Decomposition (MVMD), and informative intrinsic mode functions (IMFs) that capture temporal patterns were extracted.
- A Temporal Fusion Transformer (TFT) was trained on the generated IMFs to capture long-term dependencies, handle heterogeneous data, and provide an interpretable attention mechanism to improve the forecasting of the model.
- Optimized the parameters of TFT through hyperparameter tuning with the GOA Optimization Algorithm to enhance the model performance and robustness.
- The performance of the model is evaluated with several error metrics.
- The proposed model is interpreted with SHAP analysis to improve the transparency of the model and to identify the impact of influential features on short-term load forecasting. The result obtained is validated with a significant statistical test.

The article is structured as follows. The proposed methodology and the methods used are illustrated in Section 2. The metrics used to evaluate the model performance are discussed in Section 3. The results and discussions are described in Section 4. Finally, the conclusion and future enhancement of the model are discussed in Section 5.

## 2 Methodology

**Figure 2:**
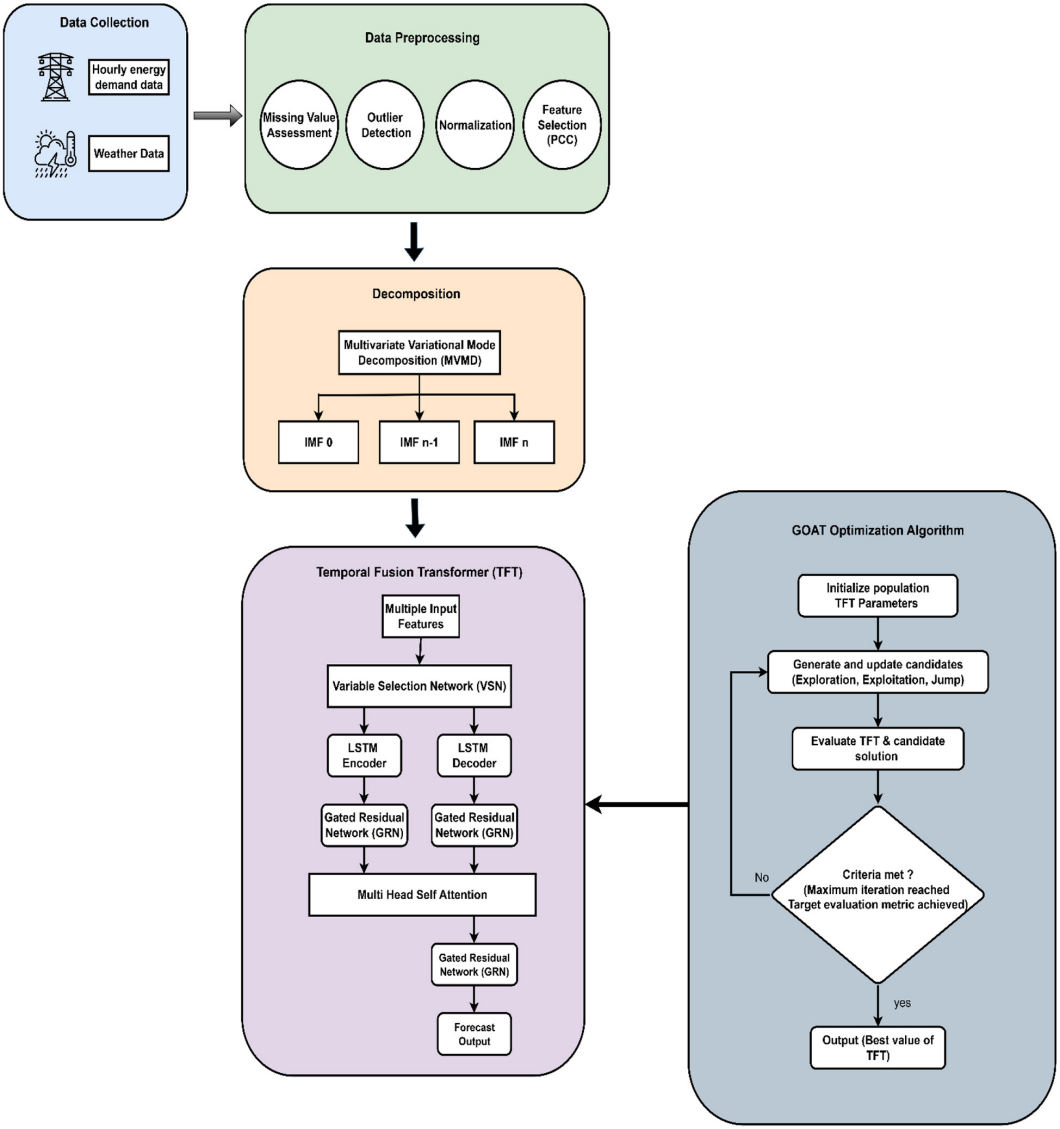
Architecture of the proposed MVMD-TFT-GOA forecasting model.

The methodologies applied in different stages of the proposed model are discussed in this section. The architecture of the proposed model is depicted in Figure 2. The methodologies employed in data preprocessing, decomposition, model development and training, optimization, and interpretability analysis are discussed as illustrated in the architecture. This proposed model applies MVMD, which robustly decomposes the complex, multivariate time series data into intrinsic mode functions (IMFs) that capture multi-scale variations, non-linear dynamics, and non-stationary components of the original signals. These IMFs are then combined as enhanced inputs into the Temporal Fusion Transformer. TFT efficiently captures long-range dependencies and non-linear interactions to operate on clean, meaningful components of input signals, potentially leading to more accurate forecasting and a more insightful understanding of feature contributions than using raw features. Unlike other optimizers, the GOAT Optimization Algorithm highlights exploration into effective, robust population-based search suitable for high-dimensional, noisy, non-convex hyperparameter spaces inherent in deep learning models. The SHAP is applied to integrate the black-box decision of a TFT model trained on MVMD features. Explainable AI not only reveals which feature is important but also provides deeper, granular insight into the specific oscillatory modes (IMFs) of the original features and multi-scale interpretability, enabling short-term load forecasting that would not be possible with traditional methods.

The accurate predictions of this proposed model facilitate enhanced generation schedules and refined energy trading strategies, leading to better operational efficiency and cost reduction. Grid stability is supported by precisely identifying demand fluctuations, which are crucial for renewable energy integration. SHAP interpretability expedites informed decision-making for demand-side management, enabling reliable, cost-effective energy system operations. The improvement in forecasting accuracy enables efficient energy management, reduced carbon emissions, and better renewable integration, thereby promoting the sustainability of modern power systems.

### 2.1 Data preprocessing

The data quality and reliability of the input features were ensured using data preprocessing techniques. The missing values were verified, and the numerical input features were scaled using min-max normalization. The outliers were detected using the interquartile range (IQR) method, and, using the filter-based feature selection method, the Pearson's correlation coefficient (PCC) of features with the target variable was identified. The features were ranked, and the most significant features were identified based on the linear association with the target variable (Hourly Demand Met). The outliers were detected using the IQR method. The lower bound is set to the quantile of *Q*_1_−1.5 × *IQR*, and the upper bound is set to *Q*_3_+1.5 × *IQR*, where *Q*_1_ and *Q*_3_ are considered as 25th and 75th percentiles, respectively.

The IQR outlier is represented in Equation 1 as,


$$\begin{array}{lll} \text{Interquartile range }\left(\text{IQR}\right) & = & \;Q_{3}-\;Q_{1} \end{array}$$ (1)

An outlier is detected if the data lie outside the range *Q*_1_−*k*×IQR, *Q*_3_+*k*×IQR. Where *k* = 1.5. *Q* is a quantile.

In min max normalization, the technique used to transform numerical data into a fixed range, usually 0–1, is represented in Equation 2 as,


$$\begin{array}{l} z_{\text{normalization}}=\frac{z-\;z_{\min}}{z_{\max}-\;z_{\min}} \end{array}$$ (2)

where *z* is the original value.

The linear relationship between two variables is quantified with a Pearson's correlation coefficient (PCC), and it ranges from −1 to +1, representing negative and positive correlation. The Pearson's correlation coefficient is represented in Equation 3 as,


$$\text{PCC}=\frac{s(\sum uv)-(\sum u)(\sum v)}{\sqrt{\left[s\sum u^{2}-{\left(\sum u\right)}^{2}\right]}[s\sum v^{2}{\left(\sum v\right)}^{2}]}$$ (3)

where *s* is the sample size, and *u* and *v* are variables.

### 2.2 Multivariate variational mode decomposition

The MVMD decomposition technique can handle multivariate features and is an enhanced version of Variational Mode Decomposition (VMD) (Ur Rehman and Aftab, 2019). MVMD decomposes multivariate signals into a set of intrinsic mode functions (IMFs). It directly receives multivariate signals from the input data and separates the meaningful information from multiple features. Multivariate Variational Mode Decomposition (MVMD) was applied to the most significant features in the second stage after data preprocessing.

In MVMD, let the input have *Z* features with *N* multisource modulated signals *a*. It formulates the problem of multichannel signal decomposition into shared modes and ensures that the sum reconstructs the original signal accurately. As shown in Equation 4, the multivariate signal input is represented as,


$$\begin{array}{l} y\left(m\right)=\sum_{n=1}^{N}a_{n}\left(m\right) \end{array}$$ (4)

Decomposes into *N* multivariate modes of *a*_*n*_(*m*).

Where *y*(*m*) = [*y*_1_(*m*), …, *y*_*z*_(*t*)], *a*_*n*_(*m*) = *a*_*n*, 1_(*m*), …*a*_*n, Z*_(*m*)

The bandwidth minimization in Equation 5 is given as,


$$\begin{array}{l} Cost\;function=\underset{n}{\sum }\underset{z}{\sum }||\partial_{m}\left[a_{+}^{n,z}\;\left(m\right)e^{-j\left(\theta_{n}\right)m}\right]|{|}_{2}^{2}\; \end{array}$$ (5)

where $a_{+}^{n,z}\;\left(m\right)$ represents an analytic modulated signal equivalent to channel number and mode number, *z* = 1, 2, …, *Z*, and optimization objective, $\underset{n}{\sum }a_{n,z}\left(m\right)=y_{z}\left(m\right)$.

The augmented Lagrangian function is expressed in Equation 6 as,


$$\begin{array}{l} L\left(\left\{a_{n,z}\right\},\left\{\theta_{n}\right\},{\left(\epsilon \right)}_{z}\right)=\;\beta \underset{n}{\sum }\underset{z}{\sum }||\partial_{m}\left[a_{+}^{n,z}\;e^{-j\theta_{n}m}\right]|{|}_{2}^{2}\\ \;+\underset{z}{\sum }||y_{z}\left(m\right)-\underset{n}{\sum }a_{n,z}\left(m\right)|{|}_{2}^{2}\\ \;+\underset{z}{\sum }\langle \in_{z},y_{z}\left(m\right)-\;\underset{n}{\sum }a_{n,z}\left(m\right)\rangle \end{array}$$ (6)

where β* is* the penalty parameter and ϵ is the Lagrange multiplier operator. To solve the constrained variational problem with the alternating direction method of multipliers (ADMM) for convergence.

The termination condition is given in Equation 7 below,


$$\begin{array}{l} \underset{n}{\sum }\underset{z}{\sum }\frac{||a_{n,z}^{i+1}-a_{n,z}^{i}|{|}_{2}^{2}}{||a_{n,z}^{i}|{|}_{2}^{2}}\;<\;\mu \end{array}$$ (7)

where μ is the tolerance value.

### 2.3 Temporal fusion transformer

The Temporal Fusion Transformer is a deep learning technique supporting multi-horizon time series forecasting. The TFT model improves the interpretability and feature selection of the models (Lim et al., 2021). The TFT framework integrates deep learning techniques with self-attention layers to handle long-term dependencies and global patterns in the data. The variable selection networks (VSNs) identify the relevant features from different input categories: static covariates, known input features, and past observations. The gating mechanism can dynamically control the information flow by removing the irrelevant components. Compared with other deep learning techniques, the Temporal Fusion Transformer provides interpretability through variable importance scores, temporal attention over time steps, and decomposition of model components for predictions. The prediction interval in the TFT produces quantile forecasts to estimate the prediction interval and express uncertainty for risk-aware decision-making. The model workflow includes data encoding, local processing using a deep learning technique, global modeling with multi-head self-attention layers that learn the dependencies across various time steps within the observation window, feature selection and gating, and multi-horizon output. The IMFs generated by MVMD enable the Temporal Fusion Transformer (TFT) to extract and leverage distinct temporal features across variables, thereby improving forecasting performance.

The components of the Temporal Fusion Transformer and its purpose are described below:

The input embedding for multivariate input features is given as follows:

$e_{t}^{k}$: linear ($x_{t\;}^{k}$) or embedding ($x_{t}^{k}$)

$x_{t\;}^{k}$: input variable *k* at time *t*

$e_{t}^{k}$: embedded/processed version of $x_{t}^{k}$

The embeddings are concatenated to form


$$\begin{array}{l} e_{t}^{\text{past}}\;\in \;R^{d},e_{t}^{\text{future}}\;\in \;R^{d},\;\text{and }e_{t}^{\text{static}}\;\in R^{d} \end{array}$$

The variable selection network for the relevant feature is selected at each time step for the static covariates as given in Equation 8.

For input $X_{t}\in \;R^{n}$,


$$\begin{array}{l} X_{t}^{\text{selected}}=\;\sum_{i=1}^{n}\alpha_{i}\left(z\right)g_{i}\left(X_{t}^{i}\right) \end{array}$$ (8)

where α_*i*_(*z*): Attention weight from static context *Z*, *g*_*i*_(.): transformation for variable *i*, ∑α_*i*_ = 1 applies both future- and past-time-varying variables.

The static covariate encoders are represented in Equation 9 as,


$$\text{GRN}(X)=\text{LayerNorm}(X+\text{Gate}(\text{ELU}(W_{1}X+b_{1}))$$ (9)

The gated residual network processes static embeddings and produces context vectors used in the VSN, Temporal processing layers, and attention components.

The LSTM hidden state *h*_*t*_, at time *t* is represented as given in Equation 10 below,


$$\begin{array}{l} h_{t}=\text{LSTM}\left(X_{t}^{\text{selected}},\;h_{t-1}\right) \end{array}$$ (10)

Skip connections ensure that the gradients flow through the LSTM layers. LSTM layers are used as the past encoder LSTM and future decoder LSTM.

The static enrichment is given in Equation 11 as,


$$\begin{array}{l} {\tilde{h}}_{t}=\text{GRN }\left(h_{t},\;c^{\text{static}}\right) \end{array}$$ (11)

To capture long-range dependencies using the Temporal Self-Attention mechanism represented in Equation 12 as,


$$\begin{array}{l} \text{Attention}\left(Q,K,V\right)=\text{softmax }\left(\frac{{QK}^{T}}{\sqrt{d_{k}}}\right)\;V \end{array}$$ (12)

where *Q, K*, and *V* are query, key, and value projections, respectively, from temporal hidden states. Attention is applied only to the decoder's horizon.

The gating mechanisms and skip connections are represented in Equation 13 as,


$$\text{GRN}(x,c)=\text{LayerNorm}(x+\text{Gate}(\text{ELU}(W_{1}x+W_{2}c+b))$$ (13)

To control the information flow, TFTs use GRN and gating layers. To control the flow of information the sigmoid layer is applied to the weighted sum of inputs.

The output layer is represented in Equation 14 as given below,

For point forecasts:


$$\begin{array}{l} {\hat{y}}_{t+r}=\text{Linear}\left(h_{t+\tau }\right) \end{array}$$ (14)

For probabilistic forecast the quantile regression can be represented as shown in Equation 15.


$$\begin{array}{l} {\hat{y}}_{t+r}^{\left(q\right)}=\text{Linear }\left(h_{t+\tau }\right)\forall \;\in \;\left\{0.1,0.5,0.9\right\} \end{array}$$ (15)

The loss function for quantile forecasting is represented in Equation 16 as,


$$\begin{array}{l} L_{q}\left(y,\hat{y}\right)=\max\left(q\left(y-\hat{y}\right),\left(q-1\right)\left(y-\hat{y}\right)\right) \end{array}$$ (16)

As shown in Equation 17, the total loss is represented as,


$$\begin{array}{l} L=\sum_{\text{tϵforecast }horizon}\sum_{q}L_{q}\left(y_{t},{\hat{y}}_{t}^{q}\right) \end{array}$$ (17)

A Temporal Fusion Transformer is used for 24-ahead prediction in short-term load forecasting, and weather, calendar, and derived features influenced the historical load data. The limitations of this model are its single-region focus and its generalization capability to other regions, which is uncertain. However, it is less robust to irregularities in load behavior (Huy et al., 2022). The Temporal Fusion Transformer model can achieve better performance in multi-horizon forecasting, selecting the required features and removing those that do not influence the model's prediction (Nazir et al., 2023). A model for short-term building energy consumption forecasting to enhance prediction accuracy and improve interpretability (Zheng et al., 2023). A Temporal Fusion Transformer can combine interpretability, long-term dependency, and the ability to handle multiple variables. TFT can be combined with decomposition techniques and optimization algorithms to improve the performance (Ye et al., 2024; Badhe et al., 2025). TFT excels at handling heterogeneous input and built-in explainability of model decisions. The gated residual network in TFT can limit the noise sensitivity and reduce overfitting to non-linear patterns as the model becomes more complex. The influential features and time step are identified by a variable selection network and a multi-head attention mechanism.

### 2.4 GOAT optimization algorithm (GOA)

GOAT is an efficient bio-inspired metaheuristic algorithm inspired by its adaptive behavior. To optimize the Temporal Fusion Transformer for short-term load prediction, GOA optimization is employed for its effective balance of exploration and exploitation, superior convergence speed, and high accuracy. An optimization algorithm like GOAT balances exploration and exploitation by effectively improving the model's robustness and efficiency (Nozari et al., 2025).

The steps involved in GOA are illustrated below:

Let the input have *g*(*y*) as the objective function, *Z* represents the number of GOATs, *K*_max_ is the maximum number of iterations, and the search space is the Lower bound *L*_*B*_ and Upper bound *U*_*B*_. Finally, the output is the best solution *Y*_*best*_.

The Temporal Fusion Transformer model was constructed by collecting tuning parameters and defining the search space bounds. The GOA is constructed by initializing the population *Y*_*n*_, where *n* = 1, 2, …, *N*. To randomly generate the GOAT's population (candidate solutions) within the defined search space bounds [*L*_*B*_, *U*_*B*_ ] as shown in Equation 18.


$$Y_{n}=L_{B}+(U_{B}-\;L_{B})\text{ random }(d)$$ (18)

where *random* (*d*) generates a d-dimensional vector in the range [0, 1].

The fitness *g*(*Y*_*n*_) is evaluated by computing the objective function of every GOAT to access the best solution. Identify the best solution, *Y*_*best*_. The GOAT with optimal fitness is found, which is considered the current global best.

The steps involved in updating the positions (main iterative loop) for each GOAT:

- Exploration: The GOATs are randomly moved to explore the search space, simulating grazing behavior through adaptive foraging.
- Exploitation: Adjust the position toward the current best-performing GOAT by local search and refinement through movement toward the best-performing GOAT.
- Occasionally, perform a significant random jump to escape local optima.
- Parasite Avoidance: Underperforming GOATs are removed, and new candidates are recruited to replace them to preserve diversity.

The fitness is reevaluated by recalculating the objective function for each updated GOAT. A better solution is found, and the record of the best is updated. The termination criteria were verified, and the algorithm was terminated when the maximum number of iterations, minimal improvement, or negligible population variance was reached. Otherwise, the iterative loop is repeated.

**Figure 3:**
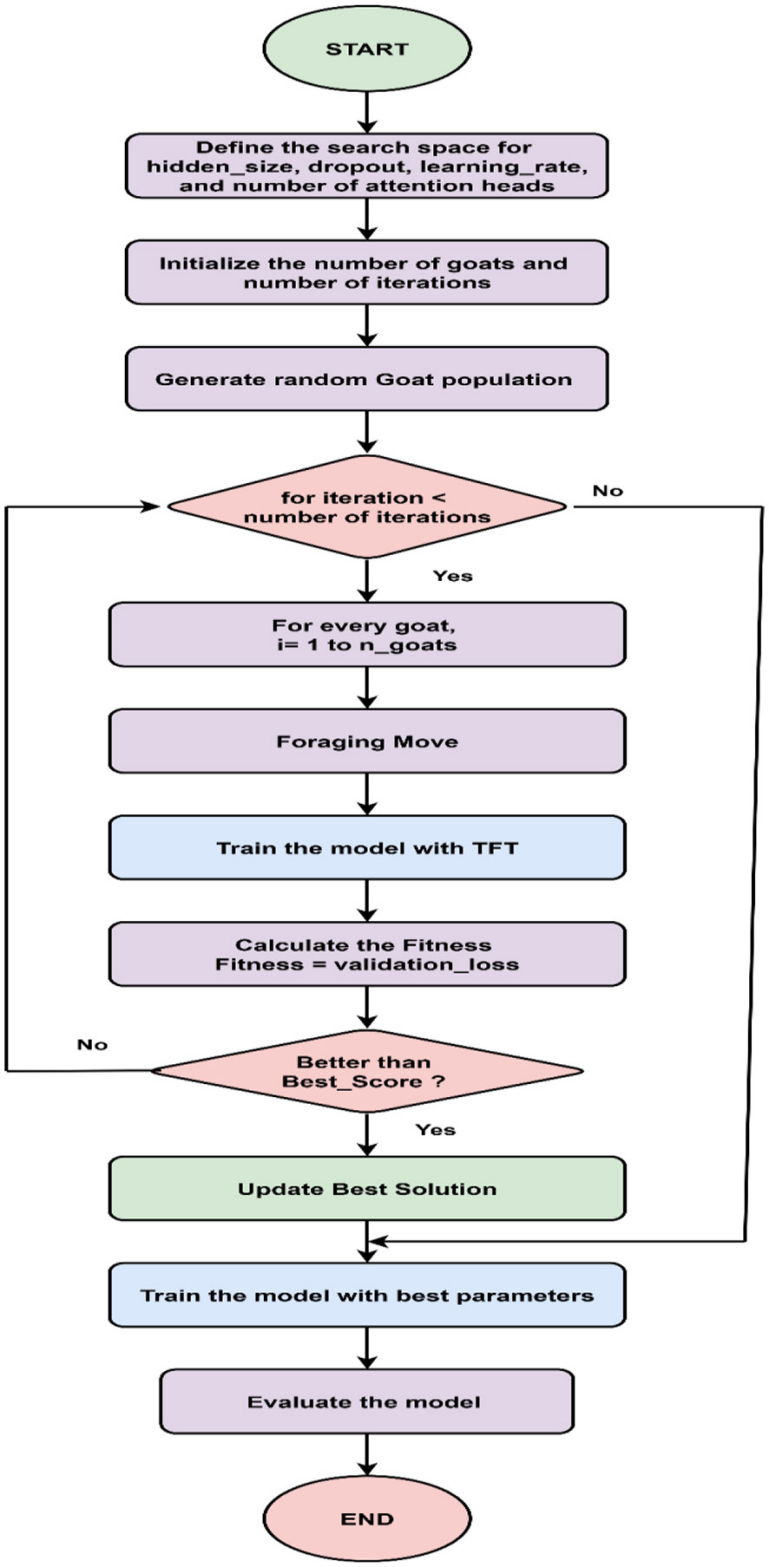
Hyperparameter tuning TFT with GOAT optimization algorithm.

TFT can handle the non-linear relationships and temporal patterns of load, calendar, and meteorological variables. However, the hyperparameter tuning, including learning rate, dropout rate, attention heads, and hidden size, is challenging and considered a global optimization problem due to its high-dimensional, non-convex, and black-box nature. The multiple-parameter interactions create many local minima, making global search strategies essential for finding the best solution. The objective of global optimization is to find the best solution over all possible solutions that encompass several local optima in a complex search space. The GOAT Optimization Algorithm is a recent meta-heuristic algorithm inspired by the herd, foraging, and climbing behavior of GOATs, aiming to reach the global minimum. Based on the best score from each iteration and random exploratory movements, this optimization algorithm effectively searches for high-dimensional hyperparameters to improve the model's performance. The benefit of hyperparameter tuning TFT with the GOAT Optimization Algorithm relies on a strong balance between exploration and exploitation. The initial iteration helps the algorithm to traverse various regions of the hyperparameter space to reduce premature convergence, and this adaptive transition supports short-term load forecasting. The challenges with metaheuristic algorithms are premature stagnation. Unlike other algorithms, GOAT maintains diversity through independent herd roaming, simultaneously increasing convergence through guided exploitation. The adaptive foraging strategy improves the robustness toward local minima. The exploration and exploitation balance can handle diverse variable search spaces and resist premature convergence. The GOAT Optimization Algorithm is effective for tuning TFT hyperparameters and improving the convergence stability. The strong adaptability toward the non-linear and dynamic nature of the demand leads to more accurate short-term load forecasting. The proposed model employs the GOA Optimization Algorithm to tune the hyperparameters of the TFT model to improve the model's performance. The model is evaluated with various performance evaluation metrics. The steps involved in hyperparameter tuning TFT with the GOA algorithm are described in Figure 3.

### 2.5 Interpretability analysis with SHapley Additive exPlanation (SHAP)

SHAP is a game-theoretic method applied in the proposed model to compute the average of each feature marginal combination across all possible features. The significant contribution of SHAP is to understand the model-specific decision in prediction and identify the global and local features that are most influential in the model's prediction. The model is retrained with the most influential features to improve its prediction performance.

## 3 Performance evaluation metrics

The model's performance is evaluated with the performance metrics, and the metrics used in this study to analyze and measure error are described below.

### 3.1 Root mean square error (RMSE)

The average of the square difference between the predicted and actual values as shown in Equation 19.


$$\begin{array}{l} \text{RMSE}=\sqrt{\frac{\sum_{j=1}^{k}{\left(A_{j}-\;P_{j}\right)}^{2}}{k}} \end{array}$$ (19)

### 3.2 Mean absolute percentage error (MAPE)

The absolute percentage difference between the actual and predicted values is used to calculate the forecasting performance as shown in Equation 20.


$$\begin{array}{l} \text{MAPE}=\frac{1}{k}\;\sum_{j=1}^{k}|\frac{A_{j}-P_{j}}{A_{j}}|\times 100\% \end{array}$$ (20)

### 3.3 Mean absolute error (MAE)

The average magnitude of the prediction error is calculated regardless of direction. This metric represents the absolute difference between the actual and predicted values as shown in Equation 21.


$$\begin{array}{l} \text{MAE}=\frac{1}{k}\sum_{j=1}^{k}|A_{j}-{\hat{P}}_{j}| \end{array}$$ (21)

### 3.4 Symmetric mean absolute percentage error (sMAPE)

The percentage between the actual and predicted value is measured symmetrically by normalizing the absolute error as shown in Equation 22.


$$\begin{array}{l} \text{sMAPE}=\frac{100\%}{k}\sum_{j=1}^{k}\frac{2|P_{j}-A_{j}|}{\left|A_{j}|+|P_{j}\right|} \end{array}$$ (22)

where *k* is the number of samples, *A*_*j*_ is the actual value, and *P*_*j*_ is the predicted value.

## 4 Results and discussions

### 4.1 Dataset description

**Figure 4:**
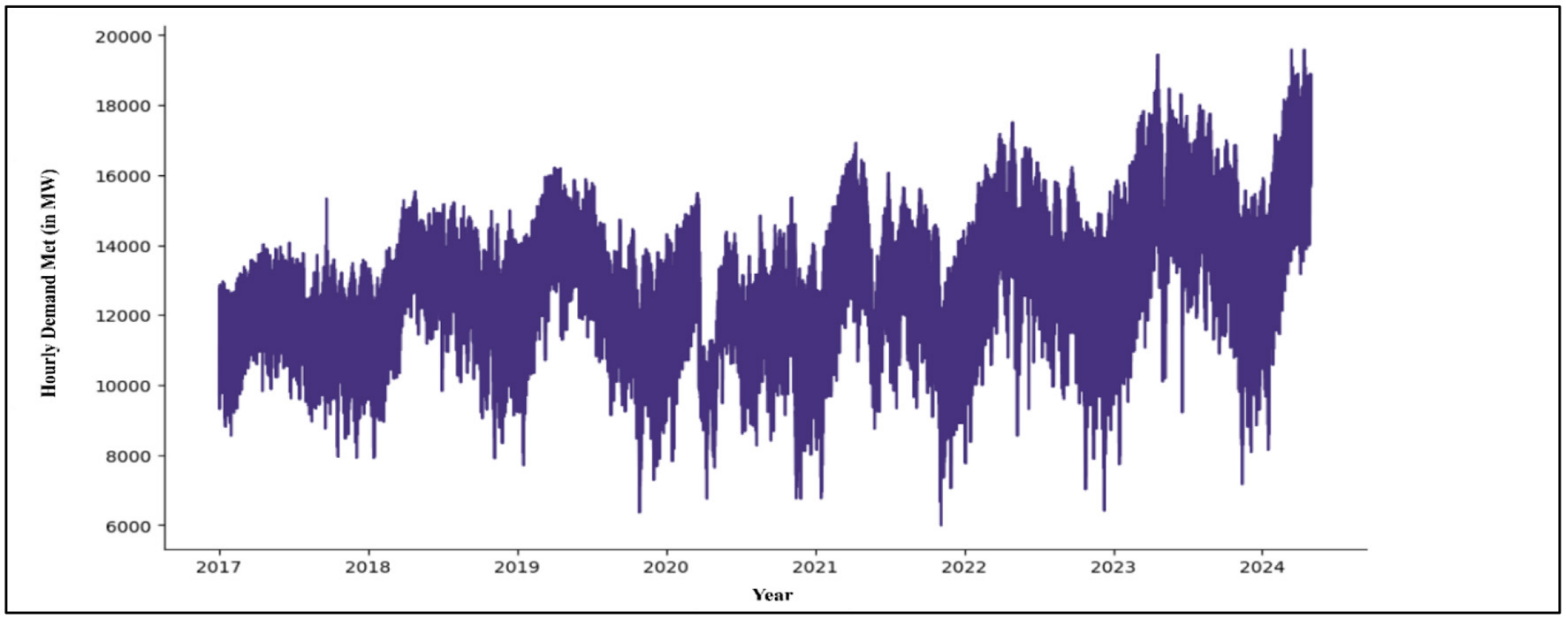
Hourly demand met (in MW) (2017–2024).

This study utilized Hourly Demand Met energy data and weather data collected from 1 January 2017 to 30 April 2024 for the state of Tamil Nadu, India. The original Hourly Demand Met (in MW) throughout the complete duration, as shown in Figure 4. The energy and meteorological features in the dataset are DateTime, Hour, Year, Day_of_weeknumber, time_idx, Is_Weekend, Hourly Demand Met, temperature_2m, wind_direction_10m, relative_humidity_2m, dew_point_2m, apparent_temperature, precipitation, rain, windspeed_10m, weathercode, and shortwave_radiation as listed in Table 2. The dataset was split into training, validation, and test data in the ratio of 80:10:10. The training, validation, and test data were 51207, 6234, and 6234, respectively.

**Table 2:** Dataset description.

Dataset attributes	Specifications
Target variable	Hourly electricity demand
Meteorological variables	Temperature, relative_humidity, dew_point, apparent_temperature, precipitation, rain, weathercode, windspeed, winddirection, and shortwave_radiation
Calendar features	Date, Day_of_week, Day_of_month, Is_Weekend
Dataset size (Instances)	64,248
Format	CSV
Time coverage	1 January 2017 to 30 April 2024
Time horizon	Hourly

The dataset was inspected to assess missing values and ensure data completeness. Feature engineering captures the temporal patterns and seasonal bias. MVMD serves as an effective feature engineering approach that handles the complex, overlapping, non-stationary trends and seasonal components in the raw data. The IMFs generated by MVMD and calendar features included in TFT can lead to robust and accurate forecasts.

The summary of the parameters and components used for Multivariate Variational Mode Decomposition, and the Temporal Fusion Transformer is presented in Table 3.

**Table 3:** Model parameter settings for MVMD and TFT in the proposed model.

Model	Parameters	Details
Multivariate variational mode decomposition (MVMD)	num_modes	3
	alpha	2000
	Tolerance	le-3
	sampling_rate	1
	Tau	0
	Init	1
Temporal fusion transformer (TFT)	Input features	Historical hourly demand met (in MW), weather features, and time-based features.
	Maximum encoder length	168 (past 7 days)
	Max prediction length	24 (next 24 h)
	Attention layer	Gated residual networks with multi-head temporal attention
	Activation	Gaussian error linear unit (GELU)
	Sequence modeling	LSTM-based encoder-decoder
	Optimizer	Adam
	Learning rate	0.0003
	Dropout	0.1
	hidden_size	32
	Attention_head_size	4
	Epochs	50
	Metrics	MAE, RMSE, MAPE, and SMAPE

### 4.2 Model development and experimental evaluation

**Figure 5:**
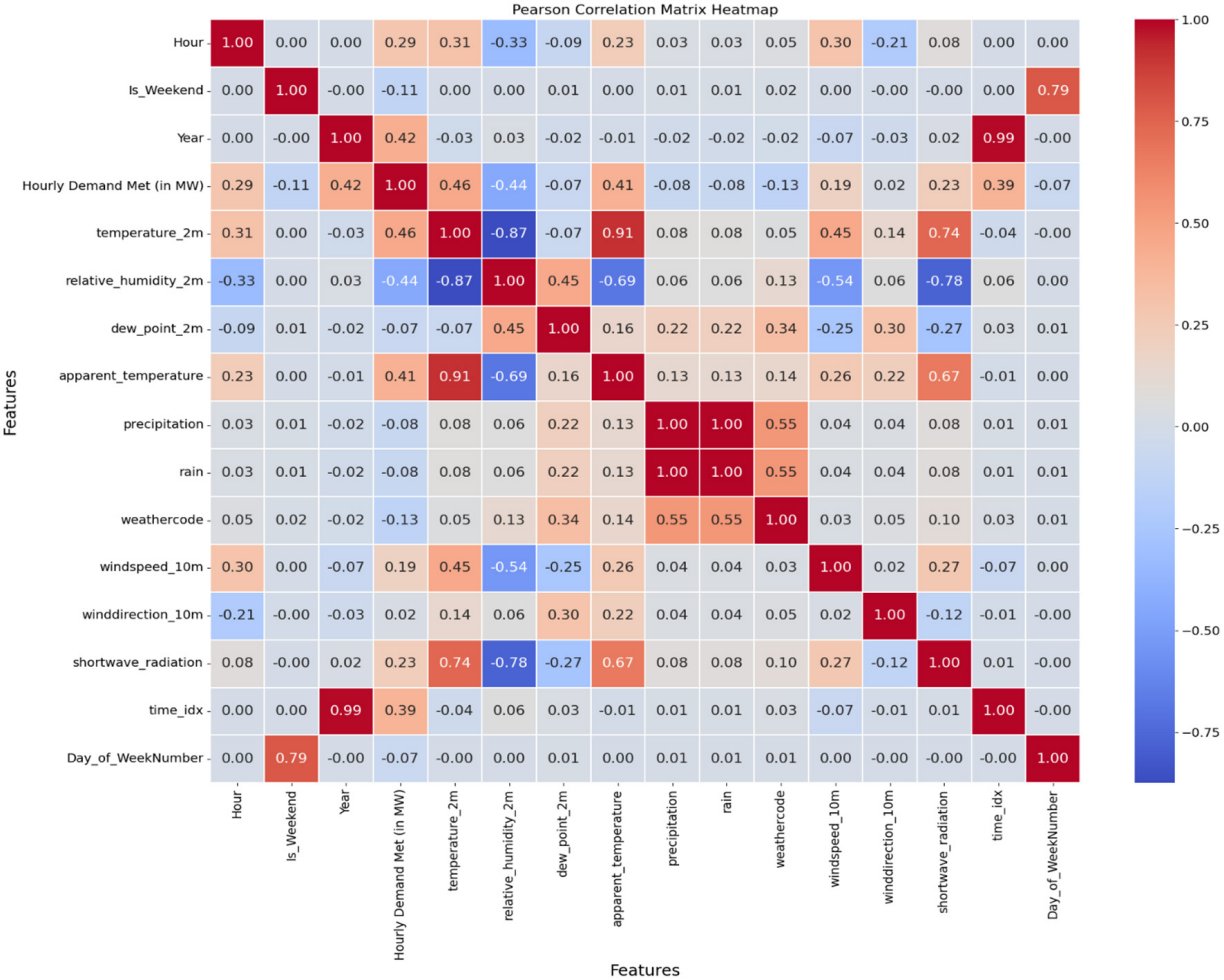
Correlation of features with the target variable.

Initially, the daily hourly load was aggregated with the weather data using timestamps and aligned with time_idx. Outliers were detected in the time series dataset for “Hourly Demand Met” (in MW) and meteorological variables to identify data points that may cause distortion. The data were normalized using min-max scaling for the range [0, 1] to ensure uniform scaling and improve convergence. The outliers were identified using the interquartile range (IQR) as the observation bounds: 1.5 × IQR for *Q*_1_ and *Q*_3_. To handle outliers, a 24 h rolling window was used to compute the median and standard deviation around every data point to identify outliers, reduce noise, and avoid extreme deviations. The correlation matrix describes the correlation between other features and the target variable, hourly demand met. The most significant features and their association with the target variable [Hourly Demand Met (in MW)] were identified using Pearson's correlation coefficient (PCC) as illustrated in Figure 5. PCC was employed for initial feature ranking due to its simplicity, speed, interpretability, and effectiveness in identifying the strong linear relationships between features. It serves as an excellent filter method for initial feature selection and computes faster than other techniques. PCC is well-suited for the preliminary assessment of high-dimensional data during early development stages. The model efficiency is improved by selecting only the highly relevant features with Pearson's correlation coefficient. Feature selection reduces redundancy and improves model generalization.

To balance the decomposition quality, the MVMD parameters are carefully selected for accuracy and computational efficiency. To avoid under- and over-decomposition, the number of modes (*k*) is set to 3, while the convergence tolerance (le-3) and uniform initialization support confirm stable convergence. A penalty factor (alpha = 2000) ensures mode mixing reduction and stable frequency separation. With noise tolerance (tau = 0) for noise-free reconstruction constraint.

The most significant 12 features selected based on the correlation with the target variable including temperature_2m (Channel 1), relative_humidity_2m (Channel 2), Year (Channel 3), time_idx (Channel 4), Hour (Channel 5), shortwave_radiation (Channel 6), windspeed_10m (Channel 7), weathercode (Channel 8), precipitation (Channel 9), Is_weekend (Channel 10), Day_of_WeekNumber (Channel 11), and winddirection_10m (Channel 12) were decomposed using Multivariate Variational Mode Decomposition to generate intrinsic mode functions (IMFs). The inter-variable dependencies are retained by decomposing the features with MVMD into frequency-based modes, thereby reducing signal complexity. The mode misalignment across features is reduced, and the MVMD adaptive decomposition supports non-stationary and non-linear multivariate signals. The Temporal Fusion Transformer was used to train and predict the model owing to its ability to handle heterogeneous features and to provide interpretable results. The modes generated for meteorological features, along with the Hourly Demand Met, are given as time-varying unknown features for TFT. To improve model performance, hyperparameter tuning was performed on the Temporal Fusion Transformer to determine optimal parameter settings. The model is trained with TFT, and hyperparameter tuning is performed with the GOAT Optimization Algorithm (GOA), a metaheuristic algorithm that works based on GOAT behaviors.

The optimal GOA parameters were determined based on practical considerations. The population size of GOATs is 5, and the number of iterations is set to 10, representing a balance between sufficiently exploring the hyperparameter search space and computational complexity. The search space includes hidden_size (32, 64), dropout (0.1, 0.3), learning rate (0.001, 0.002), and number of attention heads (2, 8), with 20 epochs to balance model complexity and generalization. This algorithm systematically searches for the optimal hyperparameter combination that minimizes a defined objective function, based on the TFT model's minimum validation loss. The best optimal hyperparameters identified were as follows: hidden size: 64, dropout: 0.1, learning_rate: 0.002, and the number of attention heads: 4. The MVMD-TFT was retrained with the optimal hyperparameter identified, and the results show that the MVMD-TFT-GOA model outperforms the other models.

**Figure 6:**
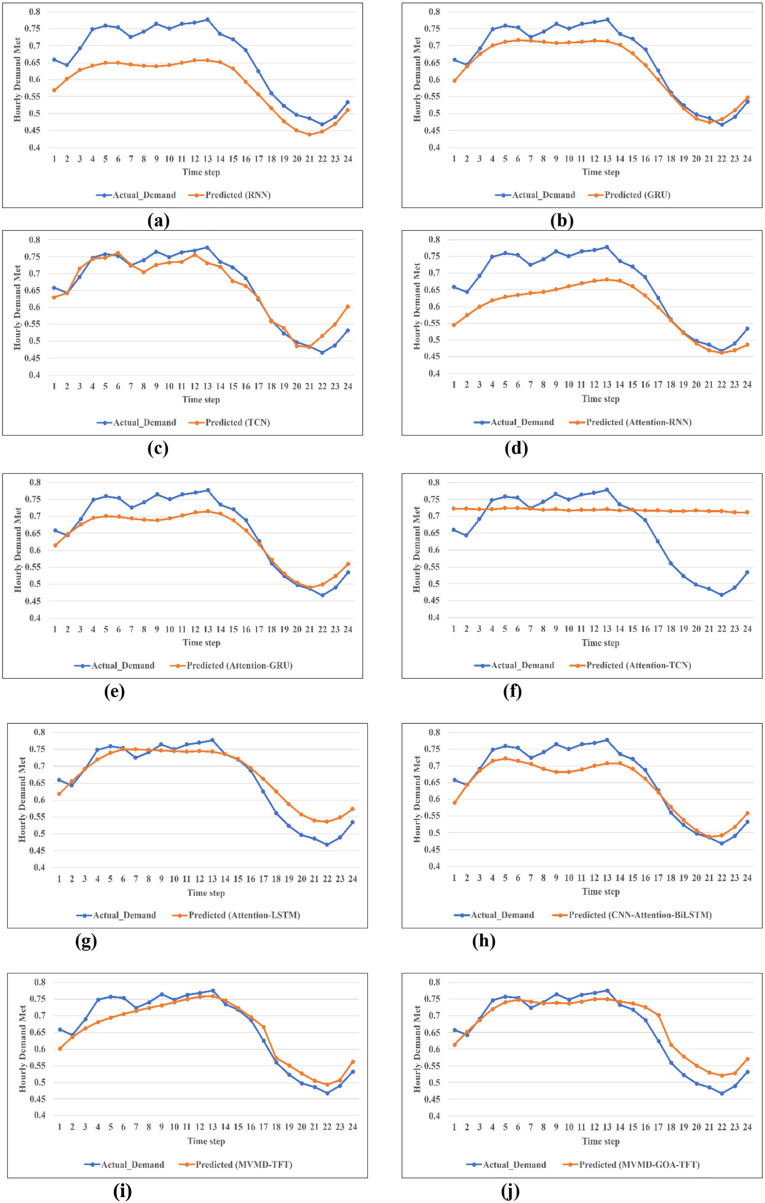
Actual vs. predicted result of the proposed MVMD-TFT-GOA model and other comparison models. **(a)** RNN. **(b)** GRU. **(c)** TCN. **(d)** Attention_RNN. **(e)** Attention_GRU. **(f)** Attention_TCN. **(g)** Attention_LSTM. **(h)** CNN_Attention_BiLSTM. **(i)** MVMD–TFT. **(j)** MVMD–TFT–GOA.

The actual vs. predicted graph for the model MVMD-TFT, MVMD-TFT-GOA, and other comparison models is given in Figure 6.

The error metrics for testing the model are evaluated with MAE, MAPE, RMSE, and sMAPE for the proposed MVMD-TFT-GOA model and other comparison models, RNN, GRU, TCN, Attention-RNN, Attention-LSTM, Attention-GRU, Attention-TCN, and CNN-Attention-BiLSTM. The measured error values for the proposed model and the comparison model are listed in Table 4. The results show that the proposed model (MVMD-TFT-GOA) improves the forecasting accuracy compared with MVMD-TFT across evaluation metrics. The comparison between MVMD-TFT-GOA and MVMD-TFT shows a 1.16% reduction in MAPE. Similarly, 4.03% reduction with RMSE, 6.41% reduction with MAE, and 6.41% reduction with sMAPE. This shows performance improvements across both absolute and relative error metrics, indicating that it not only reduces the magnitude of prediction error but also improves proportional accuracy, confirming its robustness.

**Table 4:** The performance comparison of the proposed model with other comparison models.

Models	Performance evaluation metrics			
	RMSE	MAE	MAPE (%)	sMAPE (%)
MVMD-TFT-GOA	0.0903	0.073	14.42	12.31
MVMD-TFT	0.0941	0.078	14.59	13.14
CNN-ATTN-BiLSTM	0.102	0.075	14.83	12.66
ATTN-GRU	0.1033	0.074	15.21	12.76
ATTN-LSTM	0.1055	0.077	16.23	13.29
RNN	0.1061	0.078	14.94	13.29
ATTN-RNN	0.1061	0.080	15.96	13.66
GRU	0.1064	0.077	15.21	12.89
TCN	0.1164	0.091	18.69	15.75
ATTN-TCN	0.1562	0.132	30	22.86

**Figure 7:**
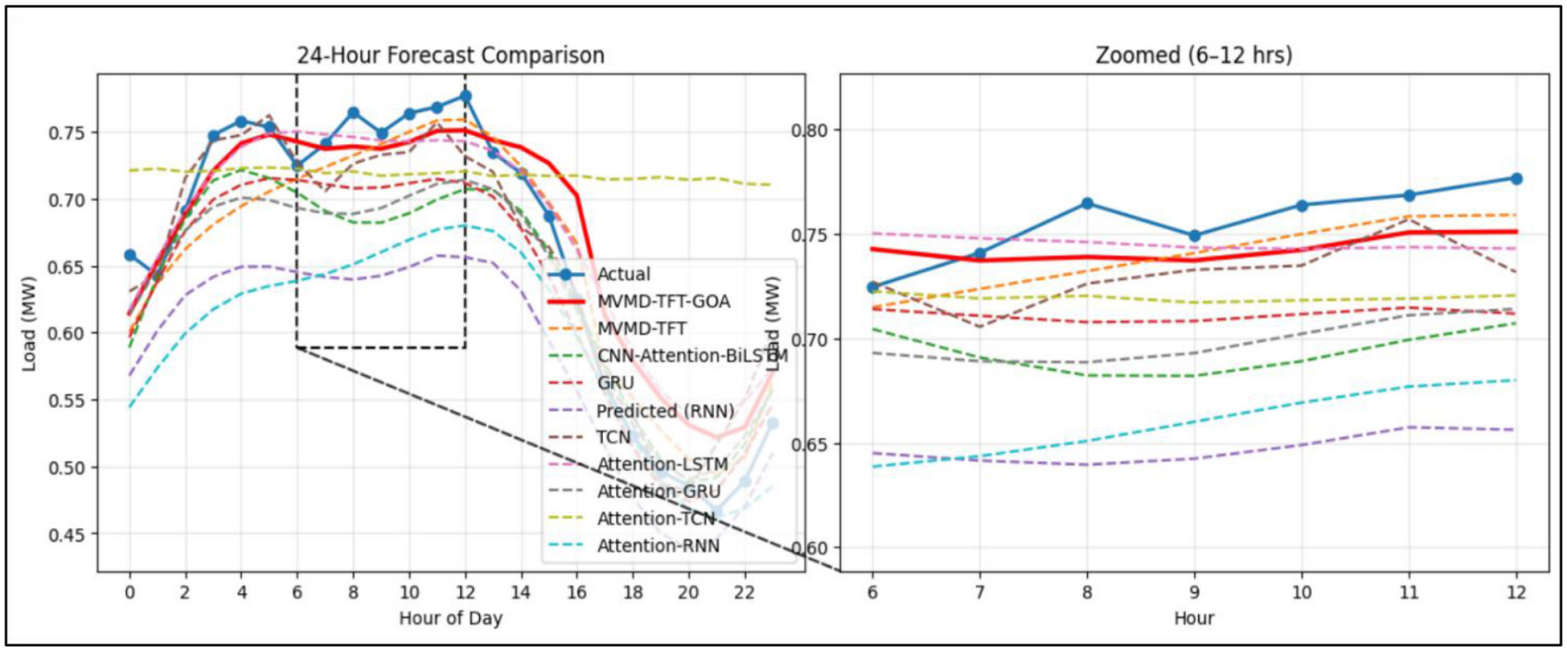
Actual vs. predicted demand over 24 h for proposed and other comparison models.

The comparison chart of the actual vs. predicted demand of the proposed model and other models for the next 24 h is depicted in Figure 7.

The GOAT optimization was selected for hyperparameter tuning based on comparison with a widely used population-based Particle Swarm Optimization (PSO). The hyperparameter search space is used similarly to the GOAT Optimization Algorithm to ensure unbiased comparison. The GOAT Optimization Algorithm (GOA) achieves lower error metrics than PSO, as shown in Table 5. GOAT achieves faster, more stable convergence, demonstrating the efficacy of hyperparameter tuning in the proposed study.

**Table 5:** Comparison of GOA with PSO.

Optimization algorithm	RMSE	MAE	MAPE	sMAPE
MVMD-TFT-GOA	0.0903	0.0732	14.42	12.31
MVMD-TFT-PSO	0.1015	0.0829	16.82	14.07

Based on the results listed in Table 5, the (MVMD-TFT-GOA) model shows better performance than MVMD-TFT-PSO. The GOA optimization is chosen for hyperparameter tuning, and the final performance of the proposed model is shown in Table 4. To strengthen the model's reliability, a statistical test using the Wilcoxon signed-rank test was conducted. The Wilcoxon signed-rank test assesses the statistical significance of the difference between the two models using a pairwise test. The forecasting error (FE_*n*_) is calculated from the *n*th forecasting values of two different models, and it is represented as, $Wilcoxon\;statistics\;\left(W_{s}\right)=\min\left\{Z^{+},Z^{-}\;\right\}$, Where *Z*^+^ represents sum_of_ranks of the First model > Second model (FE_*n*_ > 0). Similarly, *Z*^−^ represents the sum of First model < Second model (FE_n_ < 0). The Wilcoxon test was conducted at the 95% confidence interval (α = 0.05). When comparing the significance of MVMD-TFT-GOA with the conventional baseline model RNN, the *p*-value is 0.0005, and hence the null hypothesis (*H*_0_) is rejected.

**Figure 8:**
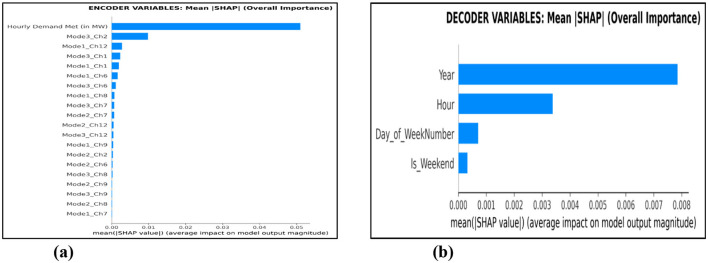
Encoder and decoder variable importance. **(a)** Encoder variable importance. **(b)** Decoder variable importance.

The interpretation of the proposed model (MVMD-TFT-GOA) is analyzed using SHAP to identify the most influential features and their impact on forecasting. The variable importance for the encoder and decoder variables is depicted in Figure 8. Each bar length represents the average magnitude of the impact of features, providing a clear understanding of encoder and decoder features that are most influential on the model's prediction. The encoder influence variables (unknown or historical features) are Hourly Demand Met, Mode3 of channel2 from relative_humidity_2m, Mode1_channel12 from winddirection_10m, Mode3 and Mode1 of channel1 from temperature_2m, Mode1 and Mode3 of channel 6 form shortwave_radiation, Mode1 of channel8 from weathercode, Mode3 and Mode 2 of channel7 from windspeed_10m, Mode2 and Mode3 of channel12 from winddirection_10m, Mode1 of channel9 from precipitation, Mode2 of channel2 from relative_humidity, and Mode2 of channel6 from shortwave_radiation. The decoder influence variables (known future features) are Year, Hour, Day_of_WeekNumber, and Is_Weekend.

From the SHAP analysis, the following features are identified as prominent features of the encoder: Hourly Demand Met, Mode3_ch2 (relative_humidity_2m), Mode1_ch12 (winddirection_10m), Mode3_ch1 and Mode1_ch1 (temperature_2m), Mode1_ch6 and Mode3_ch6 (shortwave_radiation), and Mode1_ch8 from weathercode based on the encoder variable importance. This shows that the historical patterns of Hourly Demand Met and weather conditions, when decomposed into oscillatory components, play a vital role in understanding past trends and predicting future demand. The dominant features of the decoder are year and hour, where year captures long-term trends in electricity consumption. The hour effectively identifies the hourly fluctuations that reflect the strong daily periodicity of electricity demand, with peaks during working hours. The weekly cycles and weekend consumption are also important, as they reflect consumption behavior affecting demand with different usage patterns.

The MVMD-identified significant weather features have a direct influence on seasonal patterns of electricity demand. Temperature modes can capture the effects of highly complex heating and cooling loads on the ambient temperature. An hour captures the daily patterns throughout the day based on the consumption behavior, such as morning peak time, consumption during the daytime, etc. Based on past demand, current consumption levels are strongly influencing future needs. For instance, the high-frequency component of relative_humidity Mode3_ch2 indicates that recent short-term fluctuations in humidity have a strong impact on current predictions. Based on the mean absolute SHAP values of the encoder (0.003453) and decoder (0.003064) variables, their relative importance is identified, clearly showing the significant contribution of both unknown and known features to the model's prediction.

**Figure 9:**
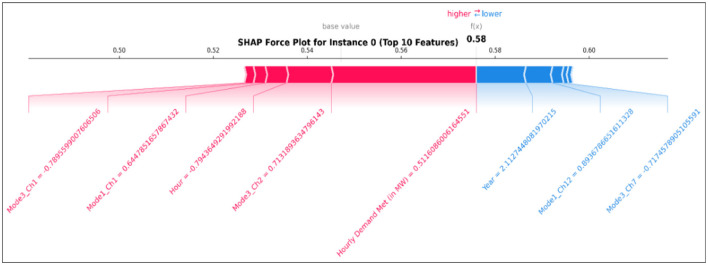
Force plot.

The SHAP force plot is illustrated in Figure 9 for model explainability, with two different colors indicating that red pushes the model prediction up and blue pushes it down. The force plot visualizes how each feature plays a vital role in the model's prediction, from the model's average across the entire dataset to the final output for a single instance. The contribution of past hourly demand data and the MVMD components, such as Mode3_Ch2 (relative_humidity), Mode1_Ch1, and Mode3_Ch1 (temperature), for instance, 0, is consistent.

From the SHAP analysis it is clear that the top-10 features have significant impact on the model prediction and those features are Hourly Demand Met (in MW), Mode3_Ch2 (relative_humidity), Mode1_Ch12 (wind_direction), Mode3_Ch1 (temperature_2m), Mode1_Ch1 (temperature_2m), Mode1_Ch6 (shortwave_radiation), Mode3_Ch6 (shortwave_radiation), Mode1_Ch8 (weathercode), Mode3_Ch7 (windspeed_10m), Mode2_Ch7 (windspeed_10m) were selected based on SHAP ranking. The proposed model is retrained with these top-10 influential features. The proposed model outperforms the other comparisons with RMSE: 0.0856, MAE: 0.0661, MAPE: 13.32, and sMAPE: 11.12. The results show that after retraining the model with the influential features, the performance improves. This finding reinforces the effectiveness of the TFT model by integrating encoder inputs such as Hourly Demand, MVMD modes, and calendar features into its forecasting. SHAP analysis detects prediction inconsistencies and supports refining features that do not influence the model's prediction to reduce forecasting error. A balanced influence between the encoder and decoder variables suggests effective learning of the model from its past information and also leveraging future deterministic factors. The interpretability provided by SHAP values enhances confidence in the TFT model's predictions. The model's reliance on highly relevant features, such as historical hourly demand and temporal indicators (hour, year, and MVMD components of weather features), directly contributes to its strong predictive performance, as shown in the results of the performance evaluation metrics. The modes of MVMD capture the underlying oscillatory components and trends in meteorological data that are highly influential for the demand prediction. This clearly provides information about the modes generated by Multivariate Variational Mode Decomposition, which strongly influences the model's predictions.

## 5 Conclusion and future enhancement

This study presents the proposed MVMD-TFT-GOA, which combines original energy Hourly Demand Met data with weather features to achieve better performance in short-term load forecasting. After data cleaning and normalization, using Pearson's correlation coefficient (PCC), the top-12 significant features are selected based on how the features are linearly related to the target variable. The Multivariate Variational Mode Decomposition is used to effectively capture the multivariate dependencies in the input sequence, combining hourly demand data with significant weather and date-related features. MVMD adapts well to changing trends and rapid fluctuations. The generated modes are provided as input to the TFT for training and validation. After training and validation, the model is tested with the best-Temporal Fusion Transformer and evaluated using the metrics MAE, RMSE, MAPE, and sMAPE. To improve the performance, the GOA is employed to search for optimal hyperparameters for TFT. After finding the best hyperparameters, TFT is trained with the best parameters, and the result shows it outperforms other models with MAE: 0.073, MAPE: 14.42, RMSE: 0.0903, and sMAPE: 12.31. The GOA algorithm dynamically finds the search direction faster and results in faster convergence than evolutionary algorithms. Finally, the results are interpreted using SHAP to ensure transparency and actionable insights. From the SHAP analysis, the top-10 influential encoder features are identified, and the model is trained with the influential features. The results obtained improve the model's performance with MAE: 0.0627, MAPE: 13.32, RMSE: 0.0856, and sMAPE: 11.12. The integration of MVMD-TFT-GOA results in better performance than other comparison models, thanks to the strength of all its components. MVMD decomposition reduces noise and captures multi-scale temporal patterns effectively by enhancing the quality of input features. The attention mechanism in TFT captures short- and long-term dependencies, focusing on all features and time steps to enhance the model's ability to learn complex, non-linear relationships. Comparing the results of MVMD-TFT and MVMD-TFT-GOA, it is evident that GOA *optimally* tunes the TFT hyperparameter to enhance convergence. Although the proposed model performs well, it has certain limitations. This model can improve generalization to other regions influenced by weather conditions and load variations. The future study will be focused on computational optimization and multi-regional validation to improve the practical applicability. As a future enhancement, a state-space model can be employed to handle long sequences better than the transformer model with linear complexity. Attention is expensive in transformer models; hence, alternative attention-free transformer models can be used.

## Data Availability

The raw data supporting the conclusions of this article will be made available by the authors, without undue reservation.
